# Supplementary figures and images for: Structural and Functional Analysis of BBA03, Borrelia burgdorferi Competitive Advantage Promoting Outer Surface Lipoprotein
Source: Pathogens. 2020 Oct 9;9(10):826. doi: 10.3390/pathogens9100826 (PMC7650648; doi:10.3390/pathogens9100826)

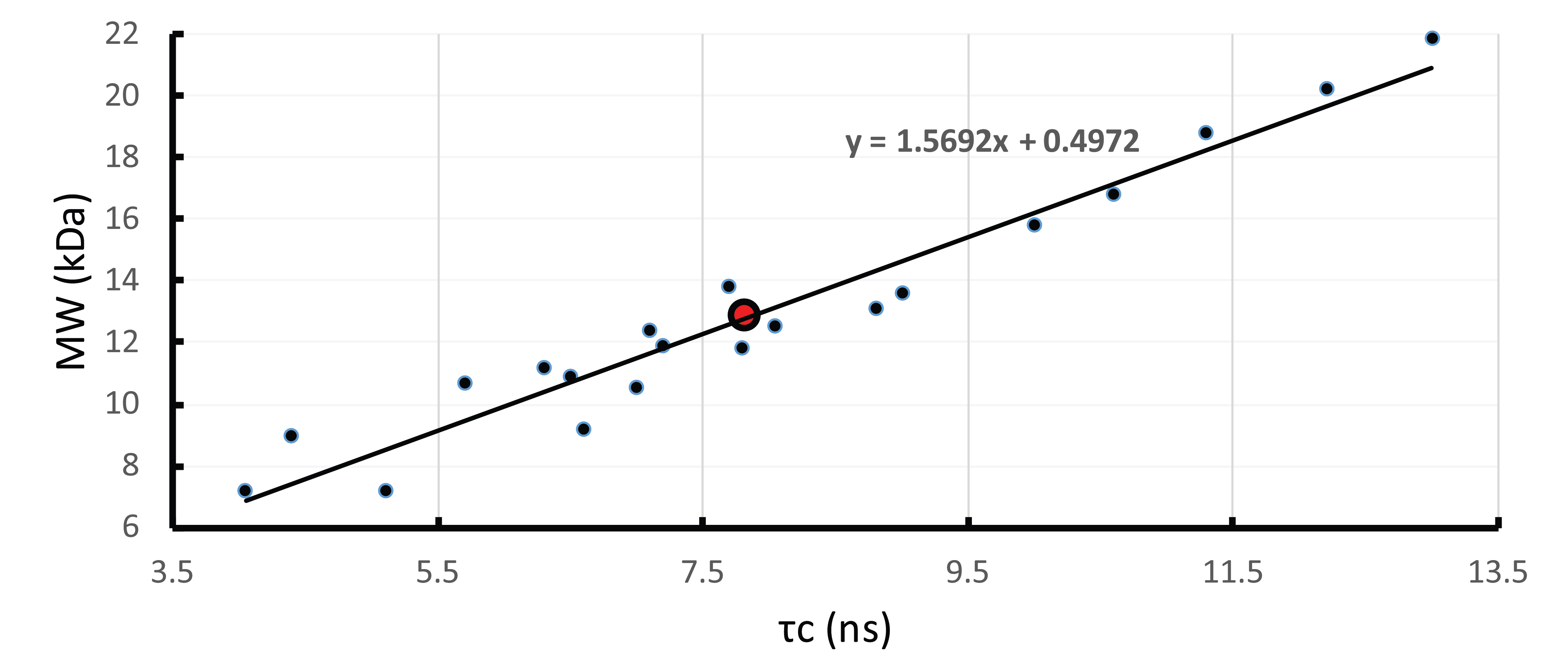

Supplement: Supplementary file 1 [file pathogens-09-00826-s001.zip › FigureS1.tif]

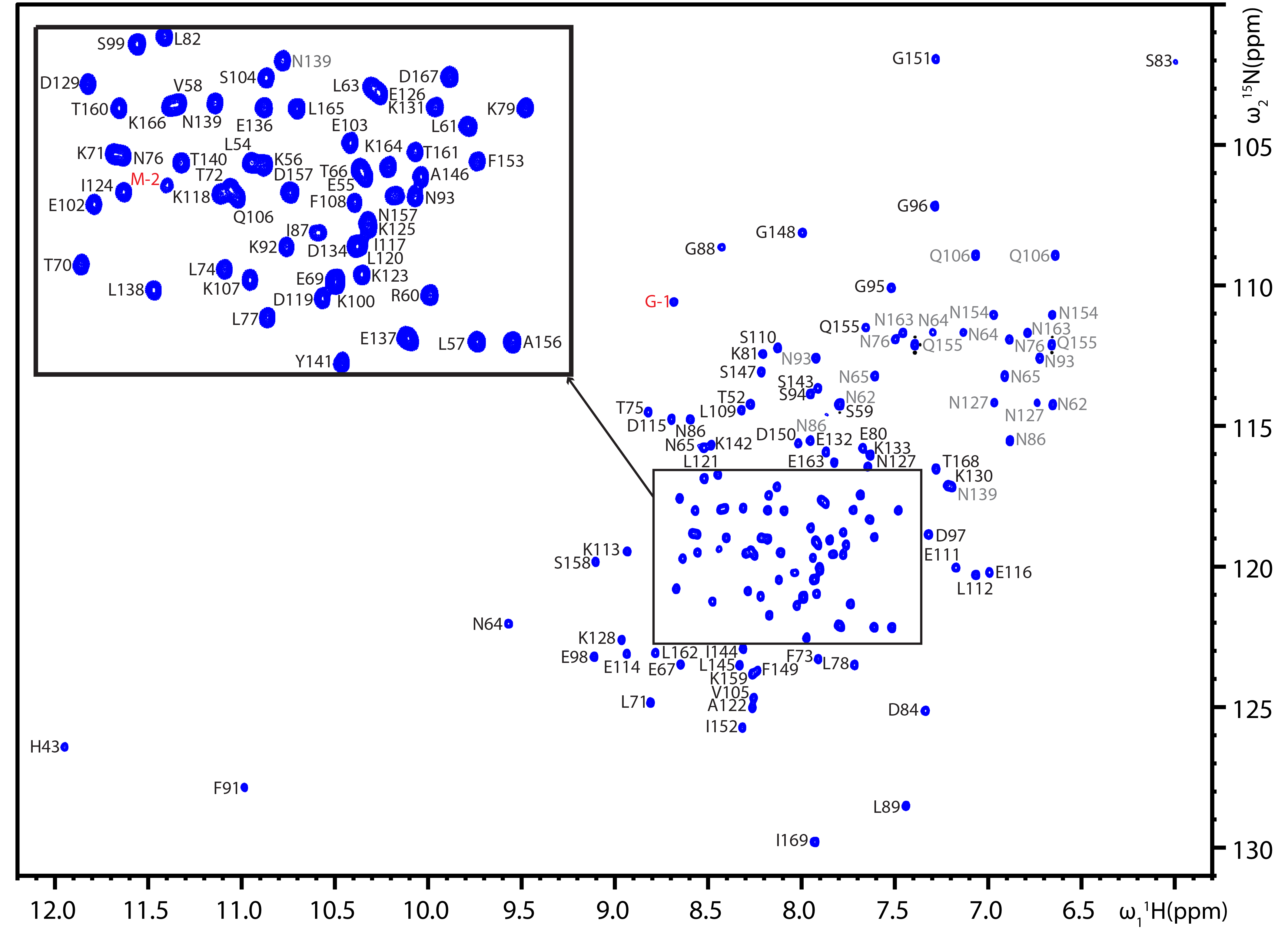

Supplement: Supplementary file 1 [file pathogens-09-00826-s001.zip › FigureS2.tif]
